# Worm Burden-Dependent Disruption of the Porcine Colon Microbiota by *Trichuris suis* Infection

**DOI:** 10.1371/journal.pone.0035470

**Published:** 2012-04-20

**Authors:** Sitao Wu, Robert W. Li, Weizhong Li, Ethiopia Beshah, Harry D. Dawson, Joseph F. Urban

**Affiliations:** 1 United States Department of Agriculture, Agricultural Research Service, Bovine Functional Genomics Laboratory, Beltsville, Maryland, United States of America; 2 Center for Research in Biological Systems, University of California San Diego, San Diego, California, United States of America; 3 United States Department of Agriculture, Agricultural Research Service, Diet, Genomics, and Immunology Laboratory, Beltsville, Maryland, United States of America; Charité-University Medicine Berlin, Germany

## Abstract

Helminth infection in pigs serves as an excellent model for the study of the interaction between human malnutrition and parasitic infection and could have important implications in human health. We had observed that pigs infected with *Trichuris suis* for 21 days showed significant changes in the proximal colon microbiota. In this study, interactions between worm burden and severity of disruptions to the microbial composition and metabolic potentials in the porcine proximal colon microbiota were investigated using metagenomic tools. Pigs were infected by a single dose of *T. suis* eggs for 53 days. Among infected pigs, two cohorts were differentiated that either had adult worms or were worm-free. Infection resulted in a significant change in the abundance of approximately 13% of genera detected in the proximal colon microbiota regardless of worm status, suggesting a relatively persistent change over time in the microbiota due to the initial infection. A significant reduction in the abundance of *Fibrobacter* and *Ruminococcus* indicated a change in the fibrolytic capacity of the colon microbiota in *T. suis* infected pigs. In addition, ∼10% of identified KEGG pathways were affected by infection, including ABC transporters, peptidoglycan biosynthesis, and lipopolysaccharide biosynthesis as well as α-linolenic acid metabolism. *Trichuris suis* infection modulated host immunity to *Campylobacter* because there was a 3-fold increase in the relative abundance in the colon microbiota of infected pigs with worms compared to naïve controls, but a 3-fold reduction in worm-free infected pigs compared to controls. The level of pathology observed in infected pigs with worms compared to worm-free infected pigs may relate to the local host response because expression of several Th2-related genes were enhanced in infected pigs with worms versus those worm-free. Our findings provided insight into the dynamics of the proximal colon microbiota in pigs in response to *T. suis* infection.

## Introduction

Swine have been widely used as a model for human diseases due to anatomic, physiological, and immunological similarities between the two species [1]. Moreover, the biodiversity of the gut microbiota between pigs and humans is comparable [2], [3]. Diverse genetic resources in pigs are readily available, which frequently leads to a whole spectrum of phenotypic changes in response to infection with bacteria, viruses, and parasites common to humans as well as similar dietary patterns. For example, Ossabaw miniature pigs respond rapidly to high-fat, high-cholesterol atherogenic diets and display numerous classical characteristics of human metabolic syndrome [4] that are modulated by daily feeding of probiotics (Solano-Aguilar et al. personal communications). Likewise, helminth infections are common in all pig production systems around the world [5] and prevalent in humans from resource poor areas worldwide. The whipworm *Trichuris suis* in pigs is an example of a common helminth infection that results in generally mild symptoms, such as diarrhea, anorexia, and retarded growth commonly controlled by management and anthelmintic drugs, but is a re-emerging problem especially in organic and free-range pig production systems. Studies on *T. suis* infection in pigs have important implications to human health because they can be zoonotic [5] and therapeutic [6]. Morphological and biometric parameters between *T. suis* and *T. trichiura* overlap and cannot be differentiated. The latter infects approximately 1049 million people globally [7]. Evolutionary relatedness and similar predilection sites in the mucosa of the upper large intestine of both species suggest that the pig-*T. suis* system can serve as an excellent model of human malnutrition and parasitic infection [8]. Recently, the immune modulating properties of helminths have been exploited to treat autoimmune diseases including inflammatory bowel diseases (IBD) such as Crohn's disease (CD) [9] and ulcerative colitis (UC) [6]. The appeal of one therapeutic agent to manage diseases as diverse as allergy, multiple sclerosis, rheumatoid arthritis, psoriatic arthritis, and autism is a powerful stimulator of further study to describe mechanisms of action (Human Helminth Co-infections Clinical Trials Database (www.niaid.nih.gov). While many trials have documented positive clinical outcomes, *T. suis* therapy has nevertheless drawn criticism over concerns of the invasiveness of worms on human physiology [10] as well as potential gastrointestinal side effects [11]. It is known that the enteric microbiota plays a critical role in the pathogenesis of IBD [12]. For example, enterobacteria are observed more frequently in CD than in healthy control human subjects [13]. Several studies have suggested that probiotics within the genera *Lactobacillus* and *Bifidobacterium* may have favorable impact on the treatment of patients with CD by altering the gut microbiota and modulating the host immune system [14]. Recently, we demonstrated that a 21-day *T. suis* infection in pigs induced a profound change in both microbial composition and metabolic potential in the lumen of the proximal colon [2]. Changes in abundance of *Succinivibrio* and *Mucispirillum* were associated with parasite-induced alterations in carbohydrate and amino acid metabolism and niche disruptions in mucosal pathology [2]. However, major determinants of phylogenetic and functional composition of the porcine colon microbiota remain unknown. In this study, we investigated the relationship between adult *T. suis* worm burden and changes in the pig proximal colon luminal microbiota. The results indicated that *T. suis*-induced changes in the proximal colon microbiota were similar regardless of the persistence or host clearance of adult worms. In addition, the local host mucosal response was associated with worm burden and the intensity of Th2-related and allergy/asthma associated gene expression.

## Results

### Worm burden and changes in localized inflammation

**Figure 1:**
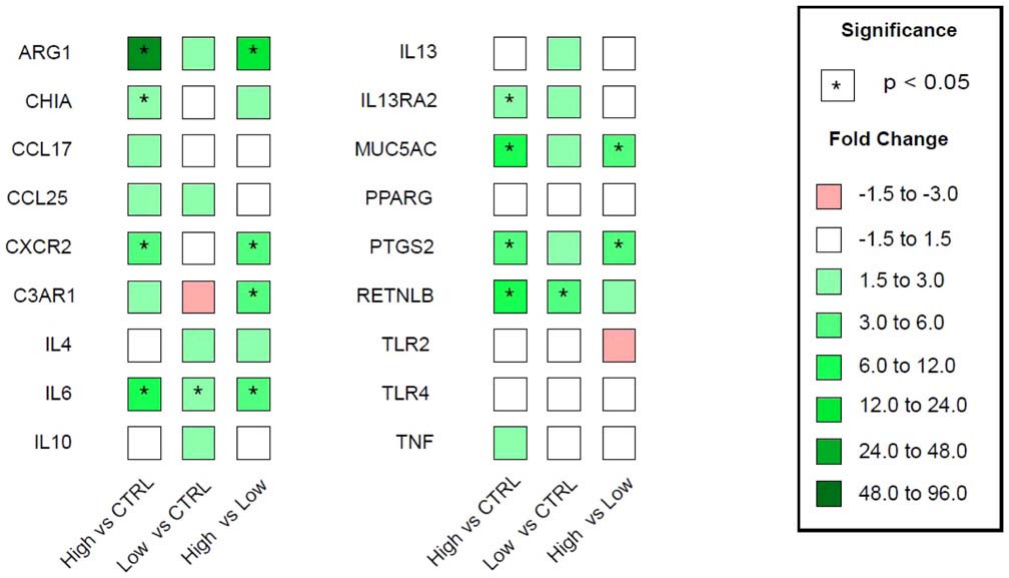
Localized changes in gene expression in the proximal colon epithelium. The relative changes in gene expression were determined by real-time RT-PCR (TaqMan) and compared between *T. suis*-infected pigs with a high worm burden (High) and low worm burden (worm-free or Low), and uninfected naïve control pigs (CTRL).

The adult *T. suis* worm burden and associated pathology in the proximal colon become more disparate in a group of out-bred pigs between seven and nine weeks after inoculation with some pigs showing fewer than 10 worms and an apparently normal mucosa and others showing hundreds of worms with localized inflammation, mucus production, and smooth muscle hypertrophy; worm clearance is virtually complete between weeks 9 and 11 [15]. We selected three infected pigs at 53 days after inoculation that had >300 adult worms (667±391 sd), five infected pigs with 0 worms, and three uninfected pigs to evaluate changes in local gene expression in the epithelial layer of the proximal colon and associated changes in the luminal microbiota. The presence of high numbers of adult worms (>300) significantly increased expression of *arg1*, *chia*, *cxcr2*, *il6*, *il13ra2*, *muc5ac*, *ptgs2*, and *retnlb* compared to uninfected control pigs and *arg1*, *cxcr2*, *c3ar1*, *il6*, *muc5ac*, and *ptgs2* were higher in infected pigs with worms versus worm-free infected pigs (Fig. 1). Expression of *ccl17*, *ccl25*, *c3ar1*, and *tnf* were not significantly higher than uninfected controls, and levels of *pprg*, *tlr2*, and *tlr4* were not increased among the groups.

### Changes in the proximal colon microbial composition in response to *T. suis* infection

**Figure 2:**
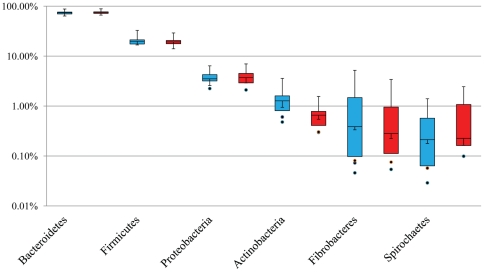
Phylum-level relative composition of the microbiome in the porcine proximal colon. Boxes denote the inter-quartile range between the 1^st^ and 3^rd^ quartiles (25 and 75%, respectively, *N* = 11). Blue: detected using MetaPhyler; Red: detected using MG-RAST. Y-axis: log scale.

Taxonomic profiles of the porcine proximal colon microbiota were evaluated using both MetaPhyler [16] and MG RAST programs [17]. The core microbiota of the porcine proximal colon included 19 phyla, 39 classes, 93 families and 121 genera, identified by the MetaPhyler method (Table 1). Of note, the 5 most abundant phyla accounted for approximately 99% of all assigned sequence reads with Bacteroidetes (72.64%), Firmicutes (20.33%) and Proteobacteria (3.69%) as among the most abundant. The percentage composition at a phylum level derived from both MetaPhyler and MG-RAST were similar (Fig. 2). In addition, our findings on the phylum-level composition using whole-genome shotgun (WGS) reads were comparable to those obtained using bar-coded pyrosequencing of the V3–V5 regions of the 16S rRNA gene [2].

**Table 1:** Taxonomic profiles of the porcine colon microbiota.

	Phylum	Class	Family	Genus
Total	27 (29)	64 (34)	213 (223)	372 (778)
Mean ±sd	23.18±1.66	52.55±5.45	159.45±16.26	238.36±28.77
	(28.09±0.30)	(34.00±0.00)	(220.18±1.25)	(661.55±18.85)
Core	19 (28)	39 (34)	93 (217)	121 (592)

Numbers of taxa identified by MetaPhyler (MG-RAST) are listed (*N* = 11).

Pigs infected with *T. suis* for 53 days had a profound change in the proximal colon microbial composition. Of 27 phyla collectively identified by MetaPhyler, Fibrobacteres, Spirochaetes, Tenericutes, and Gemmatimonadetes were significantly decreased (*P*<0.05) by infection regardless of the worm burden in the colon. The abundance of Fibrobacteres was 2.65% in the parasite naive pigs compared to 0.38% in the infected pigs while the relative abundance of Spirochaetes displayed a similar reduction from 0.79% in controls to 0.17% in infected pigs. The abundance of both phyla in the proximal colon microbiota between infected pigs with worms and worm-free was indistinguishable.

**Figure 3:**
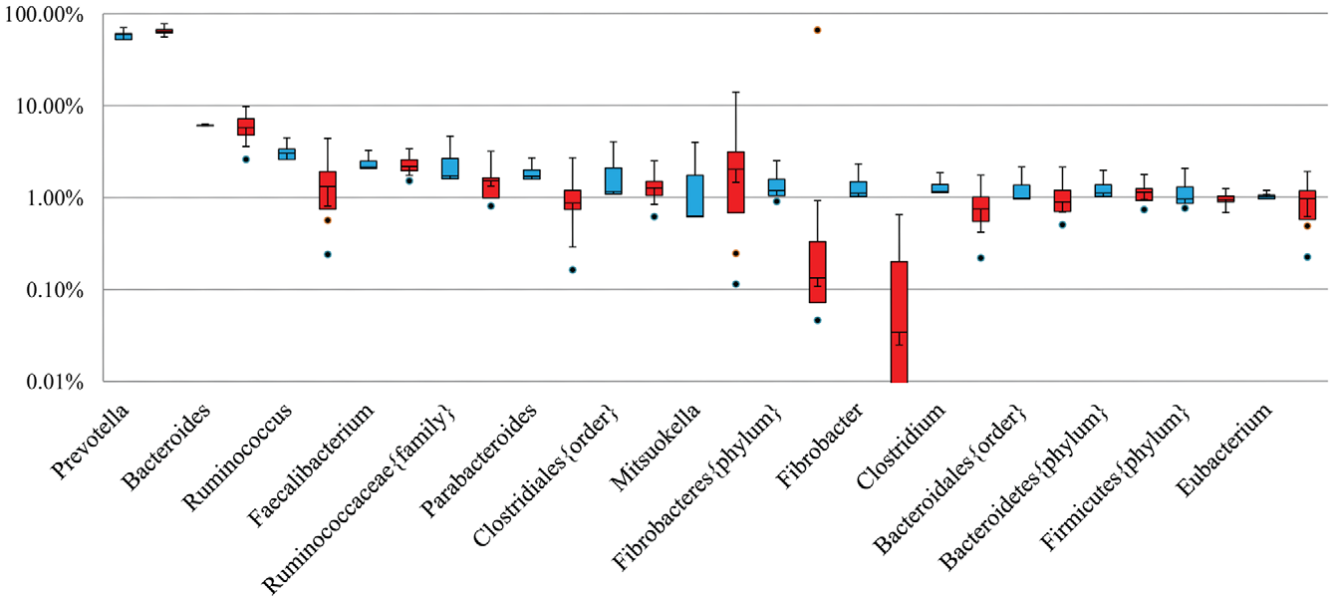
Relative abundance of 15 genera in the porcine proximal colon microbiota detected using MetaPhyler. Boxes denote the inter-quartile range between the 1^st^ and 3^rd^ quartiles (25 and 75%, respectively). Blue: Parasite naïve pigs (*N* = 3); Red: Infected pigs (*N* = 8). Symbol {} denotes a possible novel genus within the taxon indicated. For example, Ruminococcaceae{family} indicates a possible novel genus within the family Ruminococcaceae. A significant repression of relative abundance of the genus *Fibrobacter* and a possible novel genus in the phylum Fibrobacteres was detected in the proximal colon microbiota of *Trichuris suis* infected pigs. Y-axis: log scale.

The abundance of 48 of the 372 genera collectively identified in the proximal colon microbiota by MetaPhyler was significantly affected by infection. The percentage of genera altered was similar to that observed in pigs infected with *T. suis* for 21 days [2]. *Fibrobacter* and a potentially novel genus within the phylum *Fibrobacteres* were among the most abundance genera significantly affected by infection (Fig. 3). The abundance of *Treponema*, *Dorea*, and a novel genus within the phylum Spirochaetes were also significantly decreased by infection (Table 2). The abundance of *Ruminococcus* was also reduced by 2-fold at 53 days (Fig. 3) after inoculation similar to that observed at 21 days after inoculation [2]. Interestingly, the relative abundance of *Campylobacter* was low but reliably detected in the porcine proximal colon microbiota. Its abundance in infected pigs with worms was 3-fold higher than in the parasite naïve pigs (Table 2); supporting the observation that *T. suis* infection increases the risk of *Campylobacter* infection in pigs [18]. There was, however, a notable 10-fold difference in *Campylobacter* abundance in the proximal colon microbiota in infected pigs with worm compared to infected worm-free pigs (*P*<0.05). As Table 2 shows, the incidence of *Campylobacter* in infected worm-free pigs was lower than in the parasite naive pigs (*P*<0.05).

**Table 2:** Genera significantly impacted by helminth infection in the porcine colon microbiota.

Genus	Uninfected	Worm	Worm-free
Fibrobacter	2.728±1.150^a^	0.435±0.454^b^	0.420±0.488^b^
Treponema	1.468±0.195^a^	0.382±0.432^b^	0.289±0.346^b^
Spirochaeta	0.280±0.082^a^	0.080±0.072^b^	0.077±0.077^b^
Dorea	0.181±0.020^a^	0.089±0.043^b^	0.108±0.048^b^
Campylobacter	0.108±0.045^a^	0.340±0.573^a^	0.032±0.012^b^
Brachyspira	0.057±0.014^a^	0.025±0.016^b^	0.023±0.014^b^
Mycoplasma	0.028±0.004^a^	0.013±0.007^b^	0.013±0.008^b^
Thermotoga	0.024±0.008^a^	0.012±0.007^b^	0.013±0.007^a^
Actinobacillus	0.022±0.001^a^	0.014±0.004^b^	0.013±0.005^b^
Francisella	0.010±0.001^a^	0.005±0.003^b^	0.005±0.003^b^
Erysipelothrix	0.010±0.003^a^	0.005±0.003^b^	0.005±0.002^b^

Numbers denote mean ±SD of percentage composition (*N* = 4). Only genera significantly impacted detected by both MetaPhyler and MG RAST are listed. Different superscripted letters indicated significantly different at *P*<0.05 based on a modified t-test.

### The protein repertoire and pathways impacted by *T. suis* infection

Trimmed sequence reads were *de novo* assembled using SOAP_denovo_ software [19]. The process resulted in 257,415 contigs assembled at a mean length 445.02 bp (±29.77 sd) per sample (N50 = 482 bp). Genes or open reading frames (ORFs) were predicted using FragGeneScan from these contigs. These ORFs were annotated against the Pfam database (v24.0). Collectively, a total of 5,157 Pfam Protein families were identified. The ten most abundant Pfam families in the parasite naive pigs were ABC transporter (PF00005, 0.9256%), TonB dependent receptor (PF00593, 0.6344%), TonB-dependent Receptor Plug Domain (PF07715, 0.5532%), ATPase family (PF00004, 0.5281%), AcrB/AcrD/AcrF family (PF00873, 0.4989%), Glycosyl transferase family 2 (PF00535, 0.4810%), Histidine kinase-, DNA gyrase B-, and HSP90-like ATPase (PF02518, 0.4753%), Aminotransferase class I and II (PF00155, 0.4617%), Elongation factor Tu GTP binding domain (PF00009, 0.4508%), and Response regulator receiver domain (PF00072, 0.4423%). To gain insight into possible shifts in functionality and metabolic potentials in the proximal colon microbiota in response to a 53 day infection with *T. suis*, Gene Ontology (GO) terms associated with these Pfam protein families were identified. 103 of the 1390 GO terms were significantly affected by infection (*P*<0.05). As Table 3 shows, the infection seemingly had a broad impact on biological processes and molecular functions.

**Figure 4:**
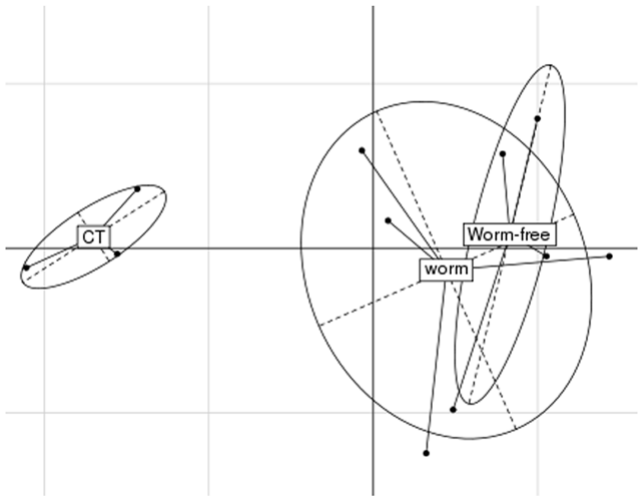
Differences in functional profiles and metabolic potentials of the porcine proximal colon microbiota between uninfected (CT) and infected groups (Worm and Worm-free). Principal component analysis (PCA) was performed using the ade4 package in R based on relative abundance of 50 selected function classes assigned using the eggNOG database.

**Figure 5:**
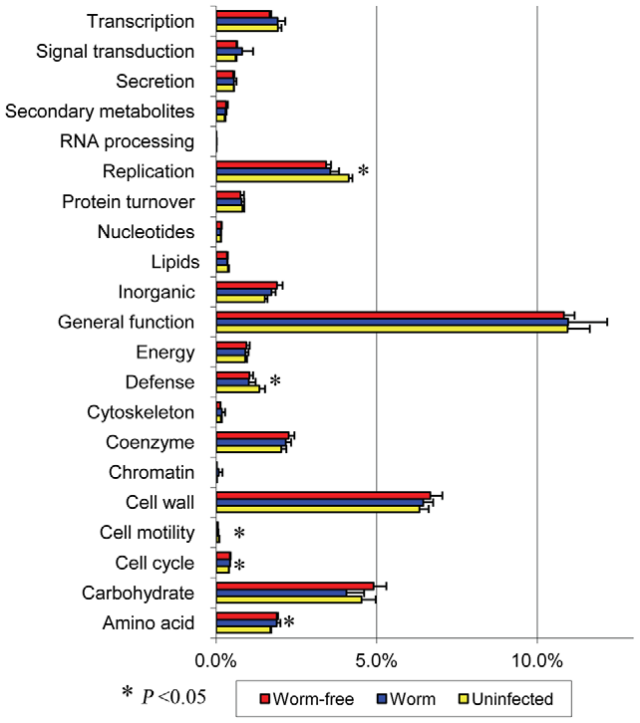
Functional categories affected by *Trichuris suis* infection in the porcine proximal colon microbiota annotated using the eggNOG database. The class labeled “unknown”, which accounted for 61.34% of hits, were not included. * denotes significantly impacted by infection, regardless of the worm burden.

The protein repertoire of the porcine colon microbiota was also assessed by the eggNOG database annotation using the MG-RAST pipeline. Of 39,777 eggNOGs identified, several proteins involved in carbohydrate metabolism were among the most abundant in the parasite naïve pigs, such as α-L-fucosidase (NOG04067, 1.05%), glycoside hydrolase family 43 (GH43, 1.01%), α-L-Rhamnosidase (NOG10735, 0.66%), and pectate lyase (NOG44882, 0.56%). Indeed, xylosidase, glucosidase, α-galactosidase, polysaccharide biosynthesis protein, and carbohydrate binding protein, as well as the above-mentioned rhamnosidase and pectate lyase, were among the most abundant in the parasite naïve pigs. *Trichuris suis* infection had a significant influence on the functional composition in the proximal colon microbiota. For example, infection induced a significant reduction in the relative abundance of α-amylase (NOG71025), from 0.14% in the control uninfected pigs to 0.04% in the infected pigs. The abundance of GH43 followed a similar trend and was significantly reduced by infection. Overall, infection resulted in a significant change in the abundance of some key eggNOGs in the proximal colon microbiota regardless of worm status, suggesting a relatively persistent change over time in the microbiota due to the initial infection (Fig. 4). Of 22 NOG functional categories identified by MG-RAST, several classes such as amino acid transport and metabolism and replication, were significantly affected (Fig. 5). In addition, the number of sequences annotated to “defense mechanisms” was significantly reduced by infection, from 1.35% in controls to 1.03% in the infected pigs regardless of worm burden.

**Figure 6:**
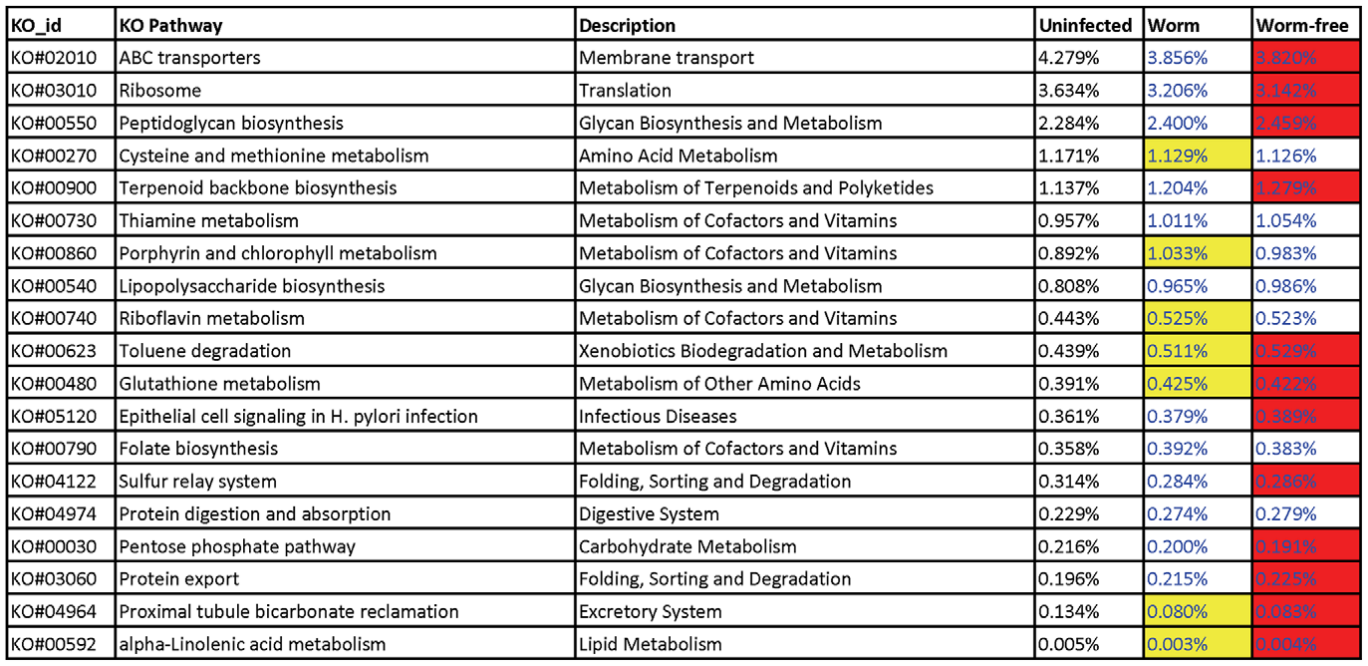
Select pathways were significantly affected infection with *Trichuris suis* in the porcine proximal colon microbiota. Numbers denote the percentage of hits positively assigned to each pathway. Different colors (Red or Yellow) indicate a significant difference between the cohorts infected with *Trichuris suis* that had adults worms (Worm) and the infected cohort where adult worms had been expelled (Worm-free). The numbers with a Blue font denote significant difference in relative abundance between uninfected control and infected (Worm + Worm-free) groups.

Metagenomic sequences were also annotated against Kyoto Encyclopedia of Genes and Genomes (KEGG) databases using the MG-RAST pipeline. A total of 7150 KEGG entries were identified. The five most abundant KEGG in the parasite naive pigs were DNA-directed RNA polymerase subunit β (K03046, 0.90%), β-glucosidase (K01188, 0.90%), carbamoyl-phosphate synthase large subunit (K01955, 0.89%), β-galactosidase (K01190, 0.77%), and excinuclease ABC subunit A (K03701, 0.73%). The relative abundance of approximately 7% of all KEGGs identified was significantly altered by infection. These KEGGs included starch phosphorylase (K00688), its abundance from 0.42% in the control uninfected pigs to 0.35% in the infected pigs. Similarly, the abundance of β-mannosidase (K01192) was also decreased as a result of infection. Approximately 10% of 297 KEGG Orthology (KO) pathways identified were affected by infection. As Fig. 6 shows, ABC transportors (KO#02010), peptidoglycan biosynthesis (KO#00550), lipopolysaccharide biosynthesis (KO#00540), alpha-linolenic acid metabolism (KO#00592) were among the 29 KO pathways affected by infection.

## Discussion

The gut microbiota plays a critical role in host nutrient metabolism as well as in the development of host immune systems [20], [21]. However, the dynamics of the gut microbiota in response to parasitic infections have only been examined recently [2], [22]. We showed previously that infection of pigs with *T. suis* for 21 days induced a profound change in proximal colon luminal microbiota with approximately ∼13% of all genera identified significantly affected by infection [2]. For example, there was a significant reduction in the relative abundance of important genera such as *Oscillibacter* and *Succinivibrio*. The changes in taxonomical profiles lead to alterations in the metabolic potential of the porcine colon microbiota, including repressing carbohydrate metabolism and lysine biosynthesis [2]. It is not clear if the worm directly altered the metabolic potential by local depletion of volatile organic compounds (VOC) that are co-factors in carbohydrate and lysine metabolism or affected metabolism upstream in the small intestine to alter the composition of metabolites in the proximal colon [2]. All pigs infected with *T. suis* for 21 days had oleic acid in the proximal colon that was not detected in parasite naïve pigs [2]. This observation suggested that the worm altered fatty acid absorption in the small intestine and that local increases in oleic acid could exert antibacterial properties to alter the local microbiome or lipolytic properties that are pro-inflammatory to the mucosa [2]. In the current study, we characterized the porcine colon microbiota at 53 days after inoculation with infective *T. suis* eggs. Specifically, we examined the effect of worm burden on the persistence of the altered proximal colon microbiota that was detected at 21 days post infection. Between seven and nine weeks after inoculation with infective *T. suis* eggs there is development of a self-cure reaction that is represented by some pigs having a persistent adult worm burden and localized inflammation, and others that have few or no adult worms and a normal mucosa [15]. This is typical of mammalian host resistance expressed as a skewed distribution of adult worm burden in genetically robust out-bred populations. While the percentage of the genera significantly affected was similar between the 21-day (13%) and 53-day infections (48 genera out of the 372 genera identified using MetaPhyler ≈13%), the difference in temporal composition profiles in the proximal colon microbiota was distinct. The most abundant genera significantly affected by a 21-day infection include *Oscillibacter* and *Succinivibrio* (both significantly decreased) as well as *Paraprevotella* (a six-fold increase in its relative abundance from 0.47% in the uninfected controls to 3.03% in the infected pigs). However, the relative abundance of these genera was not changed at 53 day post infection. The relative abundance of *Spirochaeta* and *Dorea* was significantly decreased in pigs infected with *T. suis* for 53 days, confirming the previous findings in the 21-day study [2]. Among the ten most abundant genera in the proximal colon microbiota of the parasite naive pigs (Fig. 3), *Fibrobacter* was one of the genera with its relative abundance significantly repressed by infection regardless of the worm burden (Table 2). In addition, the MetaPhyler results indicated a possible novel genus in the phylum Fibrobacteres in the porcine colon microbiota, which was also significantly reduced by infection. Sequence reads were analyzed independently using BLAT against the first sequenced genome from the phylum Fibrobacteres, *Fibrobacter succinogenes* S85 [23], a species that plays a critical role in fiber digestion in the rumen. Sequence alignment confirmed a significant reduction in abundance of *Fibrobacter* by infection. The lower level of *Fibrobacter* abundance derived from the DNA sequence alignment, compared to what was calculated from predicted protein sequences, suggests the presence of novel bacteria within the genus *Fibrobacter* or the phylum Fibrobacteres in the porcine proximal colon microbiota with sufficient sequence divergence from the S85 strain. *Fibrobacter* is known to possess a unique array of hemicellulose-degrading enzymes and is an efficient and prolific degrader of cellulose as its sole energy source [23]. The relative abundance of another group of important bacteria determining the fibrolytic capability in the rumen and hindgut, *Ruminococcus*, ranked the 3^rd^ most abundant genus in the porcine proximal colon of parasite naive pigs, showed a 2-fold reduction in response to *T. suis* infection (Fig. 3). These data indicated that the fibrolytic capacity of the proximal colon microbiota may be impaired by *T. suis* infection.

Infection of pigs with *T. suis* is associated with exacerbation of campylobacteriosis [24]–[26], which is caused by bacteria such as *Campylobacter jejuni and C*. *coli* and results in a broad range of complications, including acute diarrhea. Infections by *Campylobacter* disrupt the absorptive capacity of host epithelial cells [27]. However, *Campylobacter* does not normally cause colonic infection in pigs without a concomitant *T. suis* infection [18]. IL-4, which is strongly up-regulated by helminth infection, enhanced internalization of intestinal pig epithelial cells by *C. jejuni* and subsequent bacterial invasion in a dose-dependent manner [26], suggesting this Th2 cytokine plays a critical role in the exacerbated pathology resulting from dual infections of pigs with *T*. *suis* and *Campylobacter spp*. Localized gene expression for IL-4 was not significantly increased in the proximal colon at 53 days after inoculation (Fig. 1), but was increased earlier in the course of infection [28] which could have facilitated *Campylobacter* invasion *in situ*. In this study, we observed a 3-fold increase in the relative abundance of *Campylobacter* in the *T*. *suis* infected pigs. However, in the infected worm-free pigs *Campylobacter* abundance was significantly decreased (Table 2). The *T. suis*-facilitated uptake and antigen processing of *Campylobacter spp* by lymphoglandular complexes in the pig colon and subsequent induction of local anti- *Campylobacter* antibody responses in the ileum and colon could explain the significant reduction in *Campylobacter* from the proximal colon of worm-free infected pigs [29]. Thus, clearance of adult *T. suis* from infected pigs may have a therapeutic effect against selected bacterial pathogens that is inhibited by adult worm persistence.

**Table 3:** Gene Ontology (GO) terms significantly affected by helminth infection.

GO Term	Description	Uninfected	Worm	Worm-free
GO:0006412	translation	2.515±0.039^a^	2.285±0.108^b^	2.420±0.087^a^
GO:0005622	intracellular	1.774±0.061^a^	1.650±0.079^a^	1.647±0.053^b^
GO:0003735	structural constituent of ribosome	1.097±0.053^a^	0.928±0.059^b^	0.986±0.061^a^
GO:0005840	ribosome	1.083±0.050^a^	0.918±0.057^b^	0.973±0.061^a^
GO:0006096	glycolysis	0.339±0.004^a^	0.350±0.007^b^	0.354±0.009^b^
GO:0016769	transferase activity	0.265±0.007^a^	0.278±0.010^a^	0.286±0.007^b^
GO:0044237	cellular metabolic process	0.232±0.013^a^	0.252±0.008^b^	0.248±0.014^a^
GO:0016829	lyase activity	0.229±0.009^a^	0.213±0.008^b^	0.221±0.003^a^
GO:0009253	peptidoglycan catabolic process	0.165±0.003^a^	0.186±0.020^a^	0.187±0.008^b^
GO:0016740	transferase activity	0.125±0.006^a^	0.156±0.017^b^	0.144±0.014^a^
GO:0008484	sulfuric ester hydrolase activity	0.124±0.005^a^	0.163±0.021^b^	0.145±0.014^a^
GO:0004672	protein kinase activity	0.114±0.006^a^	0.080±0.011^b^	0.089±0.012^b^
GO:0006468	protein amino acid phosphorylation	0.102±0.007^a^	0.073±0.016^b^	0.080±0.011^b^
GO:0008134	transcription factor binding	0.082±0.008^a^	0.070±0.006^a^	0.069±0.004^b^
GO:0016620	oxidoreductase activity	0.079±0.001^a^	0.071±0.006^a^	0.072±0.004^b^
GO:0016114	terpenoid biosynthetic process	0.079±0.009^a^	0.091±0.004^a^	0.088±0.005^b^
GO:0016810	hydrolase activity [C-N bonds]	0.077±0.002^a^	0.091±0.008^b^	0.087±0.010^a^
GO:0008237	metallopeptidase activity	0.077±0.004^a^	0.088±0.001^b^	0.090±0.006^b^
GO:0009236	cobalamin biosynthetic process	0.074±0.015^a^	0.125±0.012^b^	0.103±0.013^b^
GO:0005529	sugar binding	0.074±0.005^a^	0.091±0.011^b^	0.089±0.008^b^
GO:0006526	arginine biosynthetic process	0.066±0.005^a^	0.056±0.004^b^	0.056±0.003^b^
GO:0045261	ATP synthase complex [F(1)]	0.063±0.004^a^	0.073±0.010^a^	0.076±0.005^b^
GO:0000902	cell morphogenesis	0.061±0.003^a^	0.069±0.005^a^	0.069±0.004^b^
GO:0004332	fructose-bisphosphate aldolase activity	0.060±0.002^a^	0.053±0.003^b^	0.055±0.002^b^
GO:0004356	glutamate-ammonia ligase activity	0.054±0.003^a^	0.044±0.009^a^	0.045±0.003^b^
GO:0003883	CTP synthase activity	0.050±0.001^a^	0.040±0.005^b^	0.042±0.003^b^
GO:0006221	pyrimidine nucleotide biosynthesis	0.050±0.001^a^	0.040±0.005^b^	0.042±0.003^b^

103 out of the 1390 GO terms were significantly impacted based on a modified *t*-test. GO terms with relative abundance >0.05% were listed. Numbers denote the percentage of Pfam protein families assigned to each category (mean ±sd). Different superscript letters indicate *P* <0.05.

Parasitic nematodes activate potent Th2-associated immune responses that support resistance to infection and an asthma/allergy related response that, if left uncontrolled, can contribute to mucosal inflammation [30]. Enhanced gene expression of *arg1* and *chia* represent markers of Th2-induced alternatively activated macrophages (AAM) that were diminished in the pig as the worms were cleared from the proximal colon (Fig. 1). The chemokine ligands *ccl17* and *ccl25* are related to AAM development and were increased in pigs with high numbers of worms, although not to significant levels of stimulation. The AAM plays a protective role against helminth parasites that invade the mucosa of the small intestine [31] and can regulate intestinal smooth muscle hyper-contractility in response to infection [32]. This worm-dependent modulation of AAM markers in *T. suis*-infected pigs was recently supported by the loss of expression of related markers in the proximal colon of *T. muris*-infected Balb/c mice soon after expulsion (Madden et al., personal communication). Local gene expression of *muc5ac* and *retnlb* in *T. suis*-infected pigs are related to products that contribute directly to resistance to *T. muris* in the colon of mice [33], [34]. Unregulated expression of *retnlb*, however, can also lead to mucosal inflammation during infection of mice with *T. muris* [35] as well as the expression of *ptgs2* and *c3ar1* [36] that are induced by asthma/allergy associated inflammation and were differentially expressed in pigs with high *T. suis* worm burden with increased mucosal pathology.

Epithelial cells responses to infection are protective against *T. muris* [37], [38] and the expression of *cxcr2* in *T. suis* infected pigs may relate to epithelial cell signaling as well as the expression of *il13ra2* for its role in both epithelial and smooth muscle signaling (Madden et al., personal communication) and localized control of inflammation [30], [39]. The increased gene expression of *il10* and *il13* in infected pigs that had cleared adult *T. suis*, although not statistically significant, indicated a trend toward and an anti-inflammatory response in the proximal colon that supported the appearance of a normal mucosa in these pigs.

The host ability to control infection with *T. suis* and modulate the level of localized inflammation is dependent on adult worm burden and changes in the intestinal microbiome and related metabolic changes during the course of infection [2]. The regulatory mechanisms involved are important to the rapid removal of the worm that reduces the spread of infection and reduces inflammation as well as the control of bacterial pathogens like *Campylobacter spp*. that contribute to secondary disease and represent a zoonotic threat to humans. There is also the importance of understanding these events to maximize the therapeutic potential of this nematode as a modulator of inflammatory diseases in humans. What remains is to distinguish the worm, microbiome, and host factors that skew these responses in favor of healthy outcomes.

## Materials and Methods

### Animals and parasitology

Infection protocols and sampling were essentially similar to those reported previously [2]. Briefly, 14 female piglets (Cross bred of Landrace X Yorkshire X Poland China) at three months of age were maintained indoors on sealed concretes with free access to a balanced ration and water. No antibiotics were used during the study. A single dose of infective *T. suis* eggs (2×10^4^ egg/pig) was inoculated per os (*N* = 9). The infection was allowed to progress for 53 days after inoculation. Five other pigs of the same age were orally dosed with PBS and served as parasite naive controls. All pigs were sacrificed at the same date when the infection reached 53 days. Animal management and handling were conducted based on a protocol specifically approved by the USDA-ARS Beltsville Area Animal Care and Use Committee (Protocol #10-011), following Institutional Animal Care and Use Committees (IACUC) guidelines. Luminal fecal contents were collected from the proximal colon at ∼30 cm from the ileal/caecal junction. Colon tissue samples were also collected at ∼30 cm from this junction. The pH of the contents was measured using a hand-held pH meter for semi-solid materials. Both fecal and tissue samples were snap frozen in liquid nitrogen prior to storage at −80°C until metagenomic DNA and total RNA were extracted. Colon pathology was examined by virtual and microscopic observation [18]. *Trichuris suis* worms at this stage of the infection can be visually counted on the surface of the mucosa. The pigs were free of inadvertent *Ascaris suum* infection based on the absence of worms from the small intestines and white spot lesions on the liver.

### Quantitative reverse transcriptase (RT)-PCR

Total RNA samples extracted from the epithelial cell layer of the proximal colon that was separated manually by peeling it away from the muscularis of *T. suis*-infected (three pigs with worms and five with no detected worms) and five uninfected pigs [28], including all pigs used for the microbiome study. Briefly, frozen tissue sections removed from pigs at necropsy and placed immediately in liquid nitrogen followed by storage at −80C until use. Tissues were subsequently homogenized in Trizol (Invitrogen, Grand Island, NY) and RNA was extracted from homogenized samples according to the manufacturer's instruction. The extracted RNA was treated with DNase in the presence of RNase inhibitor. RNA integrity, quantity, and genomic DNA contamination were assessed using the Experion RNA Analysis Chips (Bio-Rad). cDNA was synthesized using iScript cDNA Synthesis kit from Bio-Rad. The sequence of probes and primers and running conditions of RT-PCR were obtained from the DGIL Porcine Immunology and Nutrition Database http://www.ars.usda.gov/Services/docs.htm?docid=6065. Primers and high-performance liquid chromatography-purified, 5′,6-carboxy-4,7,2′,7′-tetrachlorofluorescein-, 3′ Black Hole Quencher-1-labeled fluorescent probes were synthesized (Biosource, Camarillo, CA). Real-time RT-PCR was performed using 15 ng/well of cDNA in 15 μl on an ABI 7900 sequence detector system (Applied Biosystems, Foster City, CA). Data for gene expression were normalized to the housekeeping gene RPL32 and converted to ΔC_T_ [40]–[41].

### Metagenomic DNA extraction and sequencing

Metagenomic DNA was extracted from fecal samples using a QIAamp DNA stool kit (Qiagen, Valenica, CA) with modifications to the protocol described [22], [42]. DNA integrity was verified using a Bioanalyzer 2100 (Agilent, Palo Alto, CA). Metagenomic DNA concentration was quantified by fluorometry. Approximately 1.0 µg of high-quality DNA was processed using an Illumina TruSeq DNA sample prep kit following manufacturer's instruction (Illumina, San Diego, CA, USA). Final individual libraries were validated, pooled based on their respective 6-bp adaptors and sequenced at 100 bp/sequence read using an Illumina HiSeq 2000 sequencer. Approximately 47,958,917±10,634,382 (mean ±sd) raw sequence reads per sample were generated for this study. Sequence reads were deposited to the MG-RAST and are publically accessible at the metagenomic analysis server (http://metagenomics.anl.gov/) (accession# 4474250.3 to 4474257.3, 4474259.3, 4474261.3, and 4474262.3).

### Data analysis and statistics

Metagenomic DNA samples extracted from the proximal colon microbiota of three parasite naïve and eight infected pigs (4 with adult worms and 4 worm-free) were sequenced. Raw sequence reads from the WGS approach were first trimmed using SolexaQA, a Perl-based software package calculating quality statistics from FASTQ files generated by Illumina sequencers [43]. Reads of host origin were then removed using Bowtie [44]. The resultant quality reads were then analyzed using MetaPhyler [16]. The relative abundance data from MetaPhyler were analyzed based on a modified *t*-test [45]

Raw sequence reads were uploaded into a MG-RAST server [46] for quantitative views of the microbial populations in the lumen of the pig proximal colon based on WGS sequence data. The data were then analyzed following the MG-RAST pipeline (v3.0) including quality filtering, dereplication to remove possible sequencing artifacts, and removal of host contaminants. Open reading frames (ORF) were then predicted using FragGeneScan [47], a recently developed program combining sequencing error models and codon usages in a hidden Markov model to improve the prediction of protein-coding region in short reads. The microbial classification was then obtained using the lowest common ancestor method in the pipeline. Sequence counts positively assigned to a given taxon at the phylum-, class-, family-, and genus- levels were normalized. Compositional differences between MetaPhyler and MG-RAST annotation platforms were analyzed using an unpaired *t*- test.

Quality WGS sequences were *de novo* assembled using SOAP*_denovo_* software [19]. ORF were predicted from all contigs greater than 200 bp using FragGeneScan (v1.14). Functional annotation was further performed according to the KEGG and Pfam (v24.0) databases. Pfam 24.0 seed alignments were downloaded, and a database of core profile HMMs was compiled using the HMMSCAN software package (v3.0), which was used to annotate predicted proteins.
